# Effect of antihypertensive medications on the risk of open-angle glaucoma

**DOI:** 10.1038/s41598-023-43420-3

**Published:** 2023-09-27

**Authors:** Jihei Sara Lee, Hye Ryeong Cha, Hyoung Won Bae, Sang Yeop Lee, Wungrak Choi, Seung Won Lee, Chan Yun Kim

**Affiliations:** 1 Department of Ophthalmology, Severance Hospital, Institute of Vision Research, Yonsei University, 50 Yonsei-Ro, Seodaemun-Gu, Seoul, 03722 Republic of Korea; 2 Department of Computer Science and Engineering, Sunkyunkwan University, Suwon, Republic of Korea; 3 Department of Ophthalmology, Yongin Severance Hospital, Yonsei University College of Medicine, Yongin-Si, Republic of Korea; 4 Department of Precision Medicine, Sungkyunkwan University School of Medicine, 2066 Seobu-ro, Jangan-Gu, Suwon, 16419 Republic of Korea

**Keywords:** Optic nerve diseases, Hypertension

## Abstract

The purpose of this study was to identify the effect of antihypertensive medication on risks of open-angle glaucoma (OAG) among patients diagnosed with hypertension (HTN). A total of 5,195 patients, who were diagnosed with HTN between January 1, 2006 and December 31, 2015, and subsequently diagnosed with OAG, were selected for analysis. For each OAG patient, 5 non-glaucomatous, hypertensive controls were matched (n = 25,975) in hypertension diagnosis date, residential area, insurance type and economic status. Antihypertensive medications were stratified into 5 types: angiotensin converting enzyme inhibitor (ACEi), angiotensin receptor blockers (ARB), calcium channel blockers (CCB), β-blockers and diuretics. Relative risks were calculated. After adjusting for age, sex, body mass index, lifestyle, comorbidities, blood pressure (BP), follow-up duration, and use of other types of antihypertensive drugs, ARB and CCB were found to slightly increase OAG risks (RR 1.1087 (95% CI 1.0293–1.1942); 1.0694 (1.0077–1.1349), respectively). Combinations of ARB with diuretics (1.0893 (1.0349–1.1466)) and CCB (1.0548 (1.0122–1.0991)) also increased OAG risks. The risks for OAG were found to increase by antihypertensive medication use, but the effects appeared to be small. Further studies are necessary to identify the associations of increased BP, medication and therapeutic effect with OAG.

## Introduction

Open-angle glaucoma (OAG) is characterized by chronic and progressive degeneration of retinal ganglion cells and associated morphologic changes to the optic nerve and retinal nerve fiber layer (RNFL)^1^. Intraocular pressure (IOP) has been identified as the single most important risk factor in development and progression of the disease, and the current treatment strategy involves reduction of IOP^2,3^. Studies have shown that IOP reduction, however, does not always slow down glaucomatous progression, particularly in normal-tension glaucoma (NTG) patients whose IOP is already low^4^. Vascular factors, such as systemic blood pressure (BP), have been implicated in the pathogenesis of NTG, instead^5,6^. However, the exact nature of the relationship between BP and OAG has remained controversial, for conflicting results have repeatedly been reported by both clinic-based and population-based studies^5,7,8^.

The relationship is further complicated by antihypertensive treatment. Some have argued that antihypertensive medications are more relevant to the pathogenesis of OAG over BP, either through overtreatment and resultant low BP^9^ or the mechanism of the drug itself^10,11^. As antihypertensive medications may potentially be modifiable risk factors for OAG, and given that each class of antihypertensive medications has its own unique mechanism, their effects on OAG risks need to be separately identified. However, previous studies have been limited by differences in the duration and severity of hypertension (HTN) to determine associations^11,12^. In the present study, using Korean National Health Insurance database, we sought to investigate OAG risks associated with individual classes of antihypertensive medication while controlling for BP, duration and concomitant use of other antihypertensive drugs.

## Results

### Baseline characteristics

Out of 31,170 subjects included in the study, 5,195 subjects were diagnosed with OAG following HTN diagnosis (Table 1). They were 62.4 ± 12.7 years old and 50.8% were males. Each OAG subject was matched to 5 controls, making up 25,975 control subjects. The glaucoma subjects consisted of older individuals (P < 0.001), and were on greater numbers of antihypertensive medications (P < 0.001). SBP was comparable between glaucoma and non-glaucoma subjects (P = 0.859), but DBP was significantly lower in glaucoma subjects (P < 0.001). Total cholesterol levels were lower, but fasting glucose levels were higher in glaucoma subjects. Follow-up durations were comparable between glaucoma and non-glaucoma subjects (P = 0.052).

**Table 1:** Baseline characteristics.

	Glaucoma	No glaucoma	*P*
	(n = 5,195)	(n = 25,975)	
Age			
40–50 years,%	721 (14.6)	6,469 (28.1)	< 0.001
51–60 years, %	1,097 (22.2)	6,931 (30.1)	
61–70 years, %	1,610 (32.5)	5,212 (22.6)	
≥ 71 years, %	1,522 (30.8)	4,450 (19.3)	
Male, %	2,641 (50.8)	13,688 (52.7)	0.014
BMI	25.4 ± 3.4	25.9 ± 3.6	< 0.001
SBP	144.1 ± 16.1	144.1 ± 16.6	0.859
DBP	87.7 ± 10.6	89.6 ± 11.1	< 0.001
Total cholesterol	220.9 ± 42.6	223.1 ± 54.4	0.003
Fasting glucose	132.6 ± 57.0	123.7 ± 45.0	< 0.001
Smoker			
Never	2,386 (55.0)	11,411 (52.6)	< 0.001
Ex	916 (21.1)	3,893 (17.5)	
Current	1,033 (23.8)	6,393 (29.5)	
Economic status, %			
1st quintile	2,747 (52.9)	13,735 (52.9)	0.999
2nd quintile	740 (14.2)	3,700 (14.2)	
3rd quintile	586 (11.3)	2,930 (11.3)	
4th quintile	605 (11.7)	3,025 (11.7)	
5th quintile	517 (10.0)	2,585 (10.0)	
Insurance type, %			
Self-employed	3,098 (59.6)	15,490 (59.6)	0.999
Employee-insured	1,744 (33.6)	8,720 (33.6)	
Medical aid	353 (6.8)	1,765 (6.8)	
No antihypertensive drug classes			
1	2,101 (40.4)	11,465 (45.1)	< 0.001
2	1,795 (34.6)	9,536 (36.7)	
3	812 (15.6)	3,386 (13.0)	
4	393 (7.6)	1,311 (5.1)	
5	94 (1.8)	277 (1.1)	
Follow-up duration, year	3 (1.5)	3 (1.5)	0.052

*P* value < 0.05 was considered statistically significant.

*BMI* body mass index, *SBP* systolic blood pressure, *DBP* diastolic blood pressure.

### History of antihypertensive medication use

Each type of medication was stratified into current, recent and past use depending on the date of most recent prescription relative to the index date (Table 2). The use of CCB and ARB either within 30 days or at the time of OAG diagnosis was associated with slightly increased OAG risks (RR 1.109 (95% CI 1.029–1.194) for ARB; 1.069 (1.008–1.135) for CCB). According to the results, past use (defined as use of a specific type of medication more than 365 days before the index date) of diuretics, CCB, ARB and ACEi was associated with decreased risks of OAG (0.931 (0.873–0.992) for diuretics, 0.878 (0.824–0.937) for CCB, 0.880 (0.798–0.971) for ACEi, and 0.874 (0.805–0.949) for ARB).

**Table 2:** Relative risks of OAG associated with the history of antihypertensive medication use.

	Glaucoma	No glaucoma	RR (95% CI)	
	(n = 5,195)	(n = 25,975)	Crude	Adjusted*
Diuretics				
Never	3,696	19,762	1	1
Current (< 31 days)	376	1,225	1.226 (1.140–1.318)	1.064 (0.979–1.156)
Recent (31–365 days)	342	1,300	1.087 (1.008–1.173)	1.033 (0.952–1.121)
Past (> 365 days)	781	3,688	0.912 (0.861–0.967)	0.931 (0.873–0.992)
β-blockers				
Never	3,766	19,799	1	1
Current (< 31 days)	379	1,465	1.127 (1.046–1.213)	1.070 (0.984–1.163)
Recent (31–365 days)	331	1,435	1.027 (0.950–1.111)	0.988 (0.906–1.077)
Past (> 365 days)	719	3,276	0.987 (0.929–1.048)	0.998 (0.935–1.065)
Calcium channel blockers				
Never	3,262	17,469	1	1
Current (< 31 days)	809	3,059	1.172 (1.110–1.236)	1.069 (1.008–1.135)
Recent (31–365 days)	475	1,912	1.115 (1.044–1.190)	1.070 (0.997–1.148)
Past (> 365 days)	649	3,535	0.869 (0.819–0.922)	0.878 (0.823–0.937)
ACE inhibitors				
Never	4,568	23,506	1	1
Current (< 31 days)	130	389	1.221 (1.083–1.376)	1.123 (0.980–1.286)
Recent (31–365 days)	141	436	1.191 (1.060–1.338)	1.042 (0.913–1.189)
Past (> 365 days)	356	1,644	0.868 (0.795–0.947)	0.880 (0.798–0.971)
ARB				
Never	3,960	20,776	1	1
Current (< 31 days)	489	1,718	1.207 (1.128–1.291)	1.109 (1.029–1.194)
Recent (31–365 days)	341	1,376	1.082 (1.001–1.169)	1.070 (0.983–1.164)
Past (> 365 days)	405	2,105	0.879 (0.816–0.946)	0.874 (0.805–0.949)

*Adjusted for age, sex, calendar year of HTN diagnosis, BMI, alcohol intake, smoking, total cholesterol, fasting glucose, SBP, DBP, MI, CAOD, CKD, follow-up duration and use of other types of antihypertensive drug.

*RR* relative risks, *CI* confidence interval, *ACE* angiotensin converting enzyme, *ARB* angiotensin receptor blocker, *HTN* hypertension, *BMI* body mass index, *SBP* systolic blood pressure, *DBP* diastolic blood pressure, *MI* myocardial infarction, *CAOD* coronary artery occlusive disease, *CKD* chronic kidney disease.

### Cumulative duration of antihypertensive medication use

The cumulative durations of antihypertensive medication use, defined as the sum of consecutive prescriptions, were divided into 2 years or less, between 2 and 4 years, and more than 4 years, as shown in Table 3. Mild increase in relative risks were noted when diuretics (1.098 (1.022–1.181)) or β-blockers (1.086 (1.014–1.164)) were used for 2 years or less. The use of diuretics for longer than 4 years was associated with decreased risks of OAG (0.874 (0.769–0.994)) relative to no use at all. Other types of antihypertensive medications did not show significant associations between cumulative duration of drug use and OAG risks.

**Table 3:** Relative risks of OAG associated with cumulative duration of antihypertensive medication use.

	Glaucoma	No glaucoma	RR (95% CI)	
	(n = 5,195)	(n = 25,975)	Crude	Adjusted*
Diuretics				
≤ 2 years	1208	4938	1.084 (1.016–1.156)	1.098 (1.022–1.181)
2–4 years	142	559	1.117 (0.994–1.256)	0.982 (0.856–1.126)
> 4 years	149	716	0.950 (0.846–1.067)	0.874 (0.769–0.994)
β-blockers				
≤ 2 years	1080	4522	1.106 (1.038–1.179)	1.086 (1.014–1.164)
2–4 years	143	720	0.951 (0.845–1.069)	0.945 (0.831–1.073)
> 4 years	206	934	1.037 (0.937–1.147)	0.982 (0.876–1.100)
Calcium channel blockers				
≤ 2 years	1223	5228	1.082 (1.028–1.140)	1.053 (0.994–1.115)
2–4 years	267	1247	1.007 (0.924–1.097)	0.945 (0.859–1.041)
> 4 years	443	2031	1.022 (0.953–1.097)	0.984 (0.909–1.065)
ACE inhibitors				
≤ 2 years	504	1956	1.088 (0.985–1.201)	1.037 (0.929–1.157)
2–4 years	64	220	1.196 (1.006–1.422)	1.021 (0.834–1.249)
> 4 years	59	293	0.890 (0.740–1.069)	0.898 (0.737–1.094)
ARB				
≤ 2 years	809	3329	1.079 (1.012–1.150)	1.004 (0.936–1.076)
2–4 years	192	840	1.027 (0.928–1.136)	1.010 (0.906–1.126)
> 4 years	234	1030	1.022 (0.930–1.122)	1.025 (0.926–1.136)

*Adjusted for age, sex, calendar year of HTN diagnosis, BMI, alcohol intake, smoking, total cholesterol, fasting glucose, SBP, DBP, MI, CAOD, CKD, follow-up duration and use of other types of antihypertensive drug.

*RR* relative risks, *CI* confidence interval, *ACE* angiotensin converting enzyme, *ARB* angiotensin receptor blocker, *HTN* hypertension, *BMI* body mass index, *SBP* systolic blood pressure, *DBP* diastolic blood pressure, *MI* myocardial infarction, *CAOD* coronary artery occlusive disease, *CKD* chronic kidney disease.

### Combination of antihypertensive medications

The effect of antihypertensive medications on OAG risks when used in combinations with other types of antihypertensive drugs was examined (Table 4). Diuretics were found to further increase OAG risks when used in combination with either β-blockers (1.094 (1.005–1.096) relative to those not on β-blockers) or ARB (1.089 (1.035–1.147) relative to those not on ARB). The OAG risks were also found to increase when CCB was used in combination with ARB (1.055 (1.012–1.099) in comparison to those not on ARB).

**Table 4:** Relative risks of OAG of antihypertensive medication stratified by combined use of other antihypertensive medications.

Other medication	Diuretics			
	No glaucoma	Glaucoma	RR (95% CI)	
			Crude	Adjusted*
Use of diuretics				
Without				
With				
Use of β-blockers				
Without	4700	1019	1	1
With	5491	1451	1.083 (1.045–1.123)	1.049 (1.005–1.096)
Use of CCB				
Without	2279	544	1	1
With	7912	1926	1.008 (0.966–1.052)	0.972 (0.924–1.023)
Use of ACEi				
Without	7908	1831	1	1
With	2283	639	1.079 (1.036–1.123)	0.984 (0.935–1.035)
Use of ARB				
Without	2648	531	1	1
With	7543	1939	1.107 (1.059–1.156)	1.089 (1.035–1.147)

Other medication	ACEi			
	No glaucoma	Glaucoma	RR (95% CI)	
			Crude	Adjusted*
Use of diuretics				
Without	1011	230	1	1
With	2627	735	1.086 (1.016–1.161)	0.985 (0.907–1.069)
Use of β-blockers				
Without	1422	346	1	1
With	2216	619	1.056 (0.996–1.120)	1.032 (0.957–1.113)
Use of CCB				
Without	957	215	1	1
With	2681	750	1.092 (1.200–1.169)	0.989 (0.910–1.076)
Use of ACEi				
Without				
With				
Use of ARB				
Without	906	177	1	1
With	2732	788	1.170 (1.087–1.260)	1.071 (0.982–1.169)

Other medication	β-blockers			
	No glaucoma	Glaucoma	RR (95% CI)	
			Crude	Adjusted*
Use of diuretics				
Without	3744	631	1	1
With	7245	1781	1.170 (1.122–1.219)	1.050 (0.999–1.104)
Use of β-blockers				
Without				
With				
Use of CCB				
Without	3026	576	1	1
With	7963	1836	1.082 (1.037–1.130)	0.996 (0.947–1.049)
Use of ACEi				
Without	8681	1772	1	1
With	2308	640	1.132 (1.087–1.178)	1.020 (0.969–1.074)
Use of ARB				
Without	3358	637	1	1
With	7631	1775	1.088 (1.044–1.134)	1.036 (0.986–1.089)

Other medication	ARB			
	No glaucoma	Glaucoma	RR (95% CI)	
			Crude	Adjusted*
Use of diuretics				
Without	4650	848	1	1
With	8947	2136	1.118 (1.078–1.159)	1.026 (0.983–1.071)
Use of β-blockers				
Without	7718	1559	1	1
With	5879	1425	1.078 (1.043–1.113)	1.010 (0.970–1.053)
Use of CCB				
Without	4012	765	1	1
With	9585	2219	1.084 (1.044–1.125)	1.037 (0.991–1.086)
Use of ACEi				
Without	11,217	2291	1	1
With	2380	693	1.153 (1.110–1.197)	1.022 (0.974–1.072)
Use of ARB				
Without				
With				

Other medication	CCB			
	No glaucoma	Glaucoma	RR (95% CI)	
			Crude	Adjusted*
Use of diuretics				
Without	5689	1064	1	1
With	10,666	2450	1.089 (1.054–1.125)	0.997 (0.959–1.036)
Use of β-blockers				
Without	8827	1754	1	1
With	7528	1760	1.069 (1.038–1.102)	1.003 (0.966–1.040)
Use of CCB				
Without				
With				
Use of ACEi				
Without	13,645	2767	1	1
With	2710	747	1.132 (1.092–1.174)	1.009 (0.964–1.056)
Use of ARB				
Without	4507	844	1	1
With	11,848	2670	1.080 (1.042–1.119)	1.055 (1.012–1.099)

*Adjusted for age, sex, calendar year of HTN diagnosis, BMI, alcohol intake, smoking, total cholesterol, fasting glucose, SBP, DBP, MI, CAOD, CKD, follow-up duration and use of other types of antihypertensive drug.

*RR* relative risks, *CI* confidence interval, *ACE* angiotensin converting enzyme, *ARB* angiotensin receptor blocker, *HTN* hypertension, *BMI* body mass index, *SBP* systolic blood pressure, *DBP* diastolic blood pressure, *MI* myocardial infarction, *CAOD* coronary artery occlusive disease, *CKD* chronic kidney disease.

## Discussion

The present study investigated the relationship between systemic antihypertensive medication and the risk of OAG by comparing the risks of OAG among HTN patients on medical therapy. According to the results, the use of CCB and ARB was associated with increased risks of OAG diagnosis, but the risks were not affected by the time length of the drug use. The effect of CCB on OAG risks were further increased if the drug was used in combination with ARB. Although the results were statistically significant, the risks mediated by antihypertensive medications in HTN patients were relatively small as indicated by low odds ratios.

Previous studies that assessed the association between antihypertensive treatment and OAG have put forth conflicting results. For instance, the Barbados Eye Study found that those who were treated for HTN had lower risks of developing OAG in comparison to those with untreated HTN^13^. Its 9-year incidence phase failed to find a significant association between antihypertensive treatment and OAG^14^, similar to the Singapore Malay Study^15^. In contrast, the Rotterdam Eye Study concluded that the low diastolic perfusion pressure increased OAG risks only in subjects receiving antihypertensive therapy^16^. Similarly, the Thessaloniki Eye Study reported that increased cupping and decreased rim area were noted in patients whose DBP fell below 90 mmHg from antihypertensive treatment, while the association was not noted in patients with similar DBP without treatment^17^. Previous studies have also failed to agree on which type of antihypertensive medications is associated with increased OAG risks. The Rotterdam Eye Study found a 1.8-fold increased risks of OAG incidence in non-glaucomatous subjects on CCB^18^. A retrospective, case–control study found significantly elevated risks of OAG diagnosis for those on CCB as well^19^. A recent meta-analysis of 10 studies on the topic also found a higher odds of glaucoma with the use of CCB^20^. Other studies, however, have shown significant associations for other types of antihypertensive medications. The same meta-analysis found β-blockers associated with lower odds of glaucoma^21^. The European Glaucoma Prevention Study noted an association between OAG development and the use of diuretics^22^, while a population-based study out of the United Kingdom found increased risks regarding the use of ACEi^19^. Another population-based, cross-sectional study by Chong et al. found increased loss of retinal ganglion cells in patients on ACEi and diuretics^23^. In contrast, a retrospective review of the Groningen Longitudinal Glaucoma Study cohort found that ARB delayed glaucoma progression in older patients and lower risk of suspect conversion in those on ACEi or ARB^24^. Many of the studies mentioned above, however, were limited in their ability to control for the duration and severity of hypertension, which may affect OAG itself. Therefore, the present study attempted to identify the effect of medications independently of HTN by matching OAG patients to those who were diagnosed with HTN at the same time. According to our analysis, the risks of OAG increased for HTN patients on CCB and ARB, after adjusting for the effect of other types of antihypertensive medications. The combinations of CCB with ARB increased the risks further in comparison to those on CCB and not on ARB.

Our results regarding CCB and ARB were in somewhat disagreement with previous cellular and animal studies on the same drugs. For instance, the components of the Renin–Angiotensin–Aldosterone (RAA) are responsible for aqueous humor formation and secretion according to studies on cultured human non-pigmented ciliary epithelial cells^25,26^. In theory, inhibition of the pathway must have beneficial effects in glaucoma patients by suppressing IOP. ARB was also found to suppress retinal ganglion cell death in an animal study^27,28^. As for calcium blockade, it is so far believed that DHP-sensitive, voltage-gated calcium channels are present on ciliary epithelial cells to regulate gap junctions between the pigmented and non-pigmented ciliary epithelial cells^29^. This way, CCB in eyes may regulate aqueous humor production and secretion by decreasing intracellular calcium influx^30,31^. Vascular smooth muscles of the choroid are also found to be regulated at least in part by calcium^32^. In a study on rabbits, oral CCB (nicardipine) enhanced retinal and choroidal blood flow^33^. Studies involving human subjects, however, have failed to yield consistent results on the ability of CCB to improve ocular circulation in glaucoma patients^29^.

More recent studies in human subjects have rather pointed the impact of CCB on glaucoma in the other direction. For instance, a meta-analysis of 11 population-based cohort studies of the European Eye Epidemiology Consortium found increased risks of glaucoma with the use of CCB^34^. This result was echoed by a recent meta-analysis that showed increased odds of glaucoma in association with CCB^20^. Both studies went on to suggest that CCBs elevate risks via mechanisms that are independent of IOP. The authors of the former study explained their finding (even though CCB is expected to reduce calcium ion levels and induce vasodilation to restore blood flow to local ischemic tissues) by suggesting that CCB-induced vasodilation may inadvertently result in diversion of blood flow from ischemic tissues to non-ischemic tissues because vasodilation is already maximized and autoregulation impaired in ischemic tissues^29,34^. If true, this mechanism may play a critical role especially in the population of the present study, which is known for its high prevalence of low-tension glaucoma. This speculation is further supported by our analysis that the combination of CCB and ARB as well as the combination of diuretics and ARB increased risks for glaucoma in comparison to those not on ARB. A previous study of BP control involving 17,187 HTN patients showed that the combination of RAS blockers with CCB or RAS blockers with CCB and diuretics resulted in better 24-h BP control, but more pronounced BP dip^35^. However, the exact mechanisms by which the medications affect glaucoma pathogenesis need to be further studied individually.

Our analysis on the association between glaucoma risks and the time lengths of antihypertensive medication yielded interesting results. When diuretics (1.098 (1.022–1.181)) or β-blockers (1.086 (1.014–1.164)) were used for 2 years or less, the risks for glaucoma increased. However, when diuretics were used for longer than 4 years, the risks of OAG (0.874 (0.769–0.994)) decreased relative to no use at all. β-blockers, in contrast, did not demonstrate significant associations with glaucoma risks when used long-term. Both β-blockers and diuretics have been associated with lower IOP when systemically administered^34^. It is possible that the such reduced IOP were not sufficient to lower glaucoma risks but delayed early detection of glaucoma instead, given that elevated IOP often draws attention to possible glaucoma patients^34^. In addition, reduction in systemic BP may have impaired perfusion of the optic nerve, causing local ischemia, thereby increasing risks in the short-term. The difference in the degree of association with glaucoma risks in the long-term between the two drugs may be related to the different patterns with which they lower BP. According to a previous study, while diuretics and β-blockers reduce systolic BP to a similar extent, β-blockers reduce diastolic BP to a greater degree^36^. The significance of diastolic BP in glaucoma has been highlighted by a number of studies in the past^37–39^. The differences between the 2 drugs to affect cardiovascular complications may have also resulted in risk differences in the long-term. For instance, diuretics have shown a more favourable outcome in preventing cardiovascular morbidity compared to beta-blockers in the past^40,41^. Furthermore, it is possible that the use of β-blockers or diuretics may be an indication of the presence of other cardiovascular comorbidities or uncontrolled hypertension, both of which may have increased risks of glaucoma^42^. We understand that this remains a possibility despite our attempt to control for most of cardiovascular comorbidities, BP levels and the severity of HTN in regression analyses. Lastly, we speculate along with the authors of a previous study that there may be a ceiling effect for some antihypertensive medications where no further increase in risks for glaucoma damage is noted once a “ceiling” has been reached^24^.

It is important to also consider that the relative inconsistency among study results, including those of the present study, may be an indication that no specific effect exists for any particular type of antihypertensive medication. Rather, HTN is a crucial risk factor and the use of antihypertensive medication is merely an indication of a history of abnormally elevated BP. HTN has consistently been suggested as a risk factor for OAG. The Blue Mountains Eye Study found that the risk of OAG increased by more than 50% if HTN is present^43^. The Baltimore Eye Survey also supported the relationship between increased OAG risks and HTN^44^. A meta-analysis from our group also demonstrated that systemic HTN increased OAG risks by approximately 20%^6^. Our previous study that investigated the association between OAG and untreated high BP demonstrated that elevated BP alone increased risks for OAG, indicating that a significant relationship exists between HTN and OAG independent of antihypertensive treatment^42^. Furthermore, CCB, which showed significant associations with OAG in multiple studies including our own, is generally not the first line treatment and is often prescribed in refractory hypertension^45,46^. Also, when adjusting for the duration of hypertension, SBP and DBP, the relative risks of those types of antihypertensive medications showing statistically significant P-values are quite small in our analysis. Their effect on OAG risks, if present, were not dose-dependent. It is likely that if there is to be any causal relationship between antihypertensive medication and OAG, the OAG risks conferred by medications are generally small and not affected by ongoing, long-term use.

The deleterious effect of overtreatment of HTN and resultant hypotension needs to be carefully assessed in the future. A number of previous studies have raised concerns about the impact of medically induced hypotension on OAG. The study as a follow-up to the Thessaloniki Eye Study found that ACE inhibitors and ARB significantly increased the likelihood of having larger cup size and higher C/D ratio^17^. However, when the analysis was repeated by separating the study population by BP levels, all classes of antihypertensive medications were associated with larger cup size and higher CD ratios for those whose DBP was below 90 mmHg while on antihypertensive treatment. The associations were not present for any of antihypertensive medications in those with DBP lower than 90 mmHg without antihypertensive medications, leading the investigators to conclude that it was the medical reduction of DBP, rather than any specific type of antihypertensive medication, that was associated with increased OAG risks. Similarly, a prescription-based study from Denmark reported that all classes of antihypertensive medications significantly increased the likelihood of a later onset of glaucoma^47^. The relationship between OAG, BP and treatment is complex and randomized clinical trials are necessary to generate strong evidence on whether and if so, what classes of antihypertensive medications are associated with OAG.

The main strengths of the present study include a large population dataset that contains a comprehensive list of potential confounding factors. Furthermore, our analysis was limited to those who do not have a history of prior antihypertensive or IOP-lowering medication use, which allowed evaluation of the incidence of glaucoma among patients with hypertension. The limitations of the study are as follows. First, the study population consists of Korean adults under a universal health insurance and screening program, and NTG is more prevalent among Korean adults, so the results may not be generalizable to other ethnic groups or populations under different health care systems. Second, the OAG cases might have been underestimated because of the asymptomatic nature in early stages, and possible omission of medical treatment due to low IOP. Third, the diagnosis and subsequent management of both OAG and HTN may not be uniform across clinicians. Fourth, prescription patterns for HTN may be complicated by concurrent systemic diseases, and the effects of other drugs or diseases may not have been completely eliminated. The number of drug types and the use of multiple drugs for the treatment of systemic HTN may be an indication for a more severe underlying condition. Fifth, prescriptions do not always reflect the actual medication use. Lastly, ocular factors such as IOP, perimetry and disc imaging were not available in this administrative claim database.

In conclusion, the use of CCB and ARB for HTN treatment was found to slightly increase OAG risks, but the risks mediated by antihypertensive medications were relatively small. Further studies are necessary to identify whether OAG risks are affected by specific mechanisms of different types of antihypertensive medications.

## Methods

### Data source

The data for the present study are derived from the Korean National Health Insurance Service-National Sample Cohort (NHIS-NSC), a population-based cohort established by the National Health Insurance Service (NHIS). The NHIS is the sole provider of healthcare in South Korea^48^. The cohort data are comprised of 1,137,861 participants, who were randomly selected from the total of approximately 46,000,000 Korean population on January 1, 2002 and followed up for 14 years until December 31, 2015. The data include disease diagnosis, drug prescriptions, interventions, and procedures as well as sociodemographic variables. The study adhered to the Declaration of Helsinki and all federal laws in the country. Informed consent was waived by the Institutional Review Board of Yonsei University Severance Hospital due to the retrospective study design and de-identified, routinely collected nature of the data. The study protocol was approved by the Institutional Review Board of Yonsei University Severance Hospital (approval number 4–2021-0689).

### Study population

**Figure 1:**
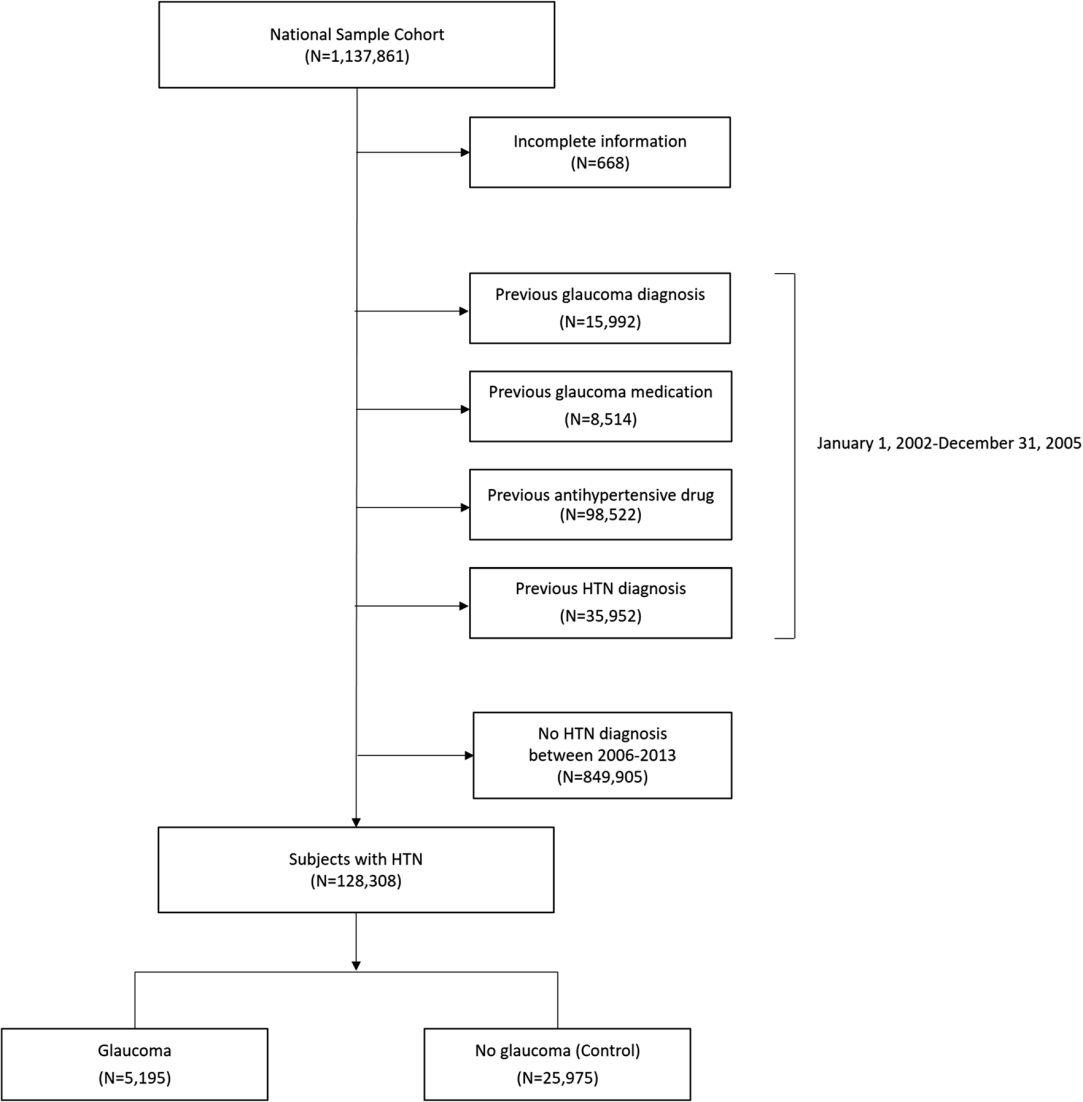
A flowchart of study population selection. From 1,137,861 subjects, those with previous glaucoma diagnosis (n = 15,992), previous glaucoma medication use (n = 8,514), previous HTN diagnosis (n = 35,952) and previous antihypertensive medication use (n = 98,522) were excluded. Subjects with HTN diagnosis were identified, and those with subsequent diagnosis of OAG were matched to 5 controls to obtain a final sample of 31,170 subjects for analysis.

The study population was selected as shown in Fig. 1, using the data of 1,137,861 adults who represent approximately 2% of Korean population. Subjects with any previous hospital claims of any form of glaucoma (n = 15,992) according to the International Classification of Diseases, 10th Revision (ICD-10) coding (H40.x, H42.x, and Q15.0) between January 2002 and December 2005 were excluded. Subjects with prior prescriptions of topical glaucoma medication (n = 8,514) during this period were also excluded. Those with any previous hospital claims of any form of HTN (n = 35,952) (I10.x, I11.x, I12.x, I13.x, and I15.x) or antihypertensive medications (n = 98,522) between January 2002 and December 2005 were excluded as well.

Subjects who were diagnosed with HTN between January 2006 and December 2015, and subsequently diagnosed with OAG were considered the cohort of the study. HTN was considered to be present if (1) hospital claims of HTN according to ICD-10 (I10.x, I11.x, I12.x, I13.x and I15.x), and (2) prescription of 1 or more antihypertensive medication for at least 30 days were both noted^49^. OAG diagnosis was defined as satisfying the following 2 criteria: (1) at least 2 outpatient/ambulatory visits between 2006 and 2015 containing ICD-10 codes of OAG (H40.10x, H40.13x, and H40.19x), and (2) prescriptions of topical glaucoma medication at least once during the same period. The first date on which OAG diagnosis criteria were satisfied was counted as the index date. One cohort subject was frequency-matched on household income, residential area and insurance type to 5 control subjects, who also satisfied HTN diagnosis criteria on the same date, leaving a final sample of 31,170 subjects for analysis. The index date for the controls was set as the index date of their matched cohort. Subjects were followed until the following endpoints: diagnosis of OAG, death, or end of the study period, whichever came first.

### Antihypertensive medication classification

Antihypertensive medications were identified in the claims data according to the protocol developed by the Korean Society of Hypertension^50^. The medications were categorized into the following 5 classes: angiotensin II receptor blockers (ARB), angiotensin converting enzyme inhibitor (ACEi), β-blockers, calcium channel blockers (CCB), and diuretics. The history of antihypertensive medication prescription was examined using the following definitions: current use (the most recent prescription either lasted until the index date or 30 days before that date), recent use (the most recent prescription ended between 31 and 365 days before the index date), and past use (the most recent prescription ended more than 365 days before the index date) and never use (no recorded use)^51^. The cumulative duration of drug use was considered only for the currently used drug at the time of the index date, and the duration was calculated by adding the time of consecutive prescriptions.

### Covariate adjustment

Individuals’ lifestyle and comorbidities were adjusted for calculation of relative risks. Subjects’ status on tobacco smoking (never, past, or current), alcohol consumption (none, 1–2 times/week, or ≥ 3 times/week), and physical exercise (none, 1–2 times/week, or ≥ 3times/week) were collected from self-reports at the time of HTN diagnosis. Body mass index (BMI), serum fasting glucose and serum total cholesterol levels were obtained during the examinations at the same time. The presence of comorbidities between the diagnosis of HTN and the endpoint, such as ischemic heart disease, myocardial infraction (MI), coronary artery obstructive disease (CAOD) and chronic kidney disease (CKD), was identified using hospital claims according to ICD-10 and considered present when at least 2 claims were made. The date of the first claim was taken as the date of diagnosis.

### Statistical analyses

Continuous variables are presented as mean ± standard deviation (SD)^52^, and categorical variables as frequency and percentage. Cox proportional hazards analyses were performed to obtain relative risks (RR) and 95% confidence intervals (CI) for OAG events for each class of antihypertensive medications. Subjects not using the antihypertensive medication in question served as the reference. The RRs were adjusted for age, sex, calendar year of HTN diagnosis, BMI, alcohol intake, smoking, total cholesterol, fasting glucose, systolic blood pressure (SBP), diastolic blood pressure (DBP), MI, CAOD, CKD, follow-up duration and use of other types of antihypertensive drug. Of note, SBP and DBP recorded at the time of HTN diagnosis were used for analyses. The variables for adjustment were selected a priori on the basis of their known associations with BP and OAG^53,54^. Statistical analyses were performed using SAS version 7.4 (SAS Institute Inc.).

## Data Availability

The data that support the findings of this study are available from the Korean National Health Insurance Service (NHIS), but restrictions apply to the availability of these data, which were used under license for the current study, and so are not publicly available. Data are however available from the authors upon reasonable request and with permission of Korean NHIS.
